# Supplementary material for: Global decline in net primary production underestimated by climate models
Source: Commun Earth Environ. 2025 Feb 1;6(1):75. doi: 10.1038/s43247-025-02051-4 (PMC11785529; doi:10.1038/s43247-025-02051-4)
Supplement: Supplementary file 2 — Supplementary Information [file 43247_2025_2051_MOESM2_ESM.pdf]

Global decline in net primary production underestimated by climate models

Thomas J. Ryan-Keogh[1*], Alessandro Tagliabue[2], Sandy J. Thomalla[1,3]

[1]Southern Ocean Carbon-Climate Observatory, CSIR, Cape Town, South Africa

[2]Department of Earth, Ocean and Ecological Sciences, School of Environmental Sciences, University of Liverpool, Liverpool, U.K.

[3]Marine and Antarctic Research Centre for Innovation and Sustainability, Department of Oceanography, University of Cape Town, Cape Town, South Africa

Author for materials and correspondence: *tryankeogh@csir.co.za

Supplementary Information

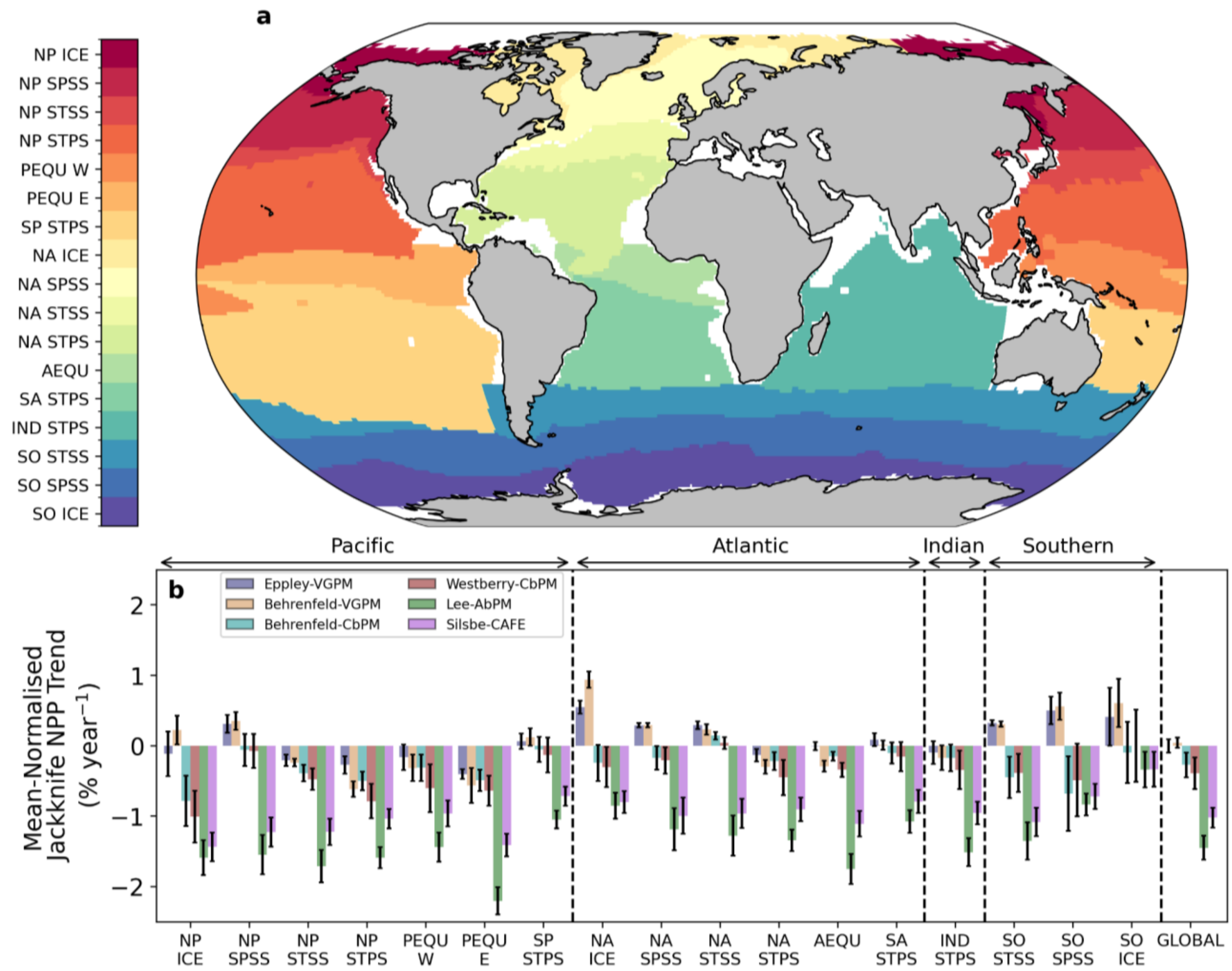

Figure S1: Comparing trends of net primary production from different remote sensing algorithms across biomes. (a) map of ocean biomes defined by Fay & McKinley[22] as the North Pacific (NP), Equatorial Pacific (PEQU), South Pacific (SP), North Atlantic (NA), Equatorial Atlantic (AEQU), South Atlantic (SA), Indian (IND) and Southern Ocean (SO) including the ice (ICE), subpolar seasonally stratified (SPSS), subtropical seasonally stratified (STSS) and subtropical permanently stratified (STPS) regions. White pixels are regions which could not be classified into a biome. (b) Bar plot of jackknife resampled area-weighted mean-normalised annual mean trends in net primary production (NPP; % year^{-1}) calculated using ordinary least squares per ocean biome for the Eppley-VGPM, Behrenfeld-VGPM, Behrenfeld-CbPM, Westberry-CbPM, Lee-AbPM and Silsbe-CAFE algorithms. Error bars represent the ±standard deviation of the jackknife resampled trends.

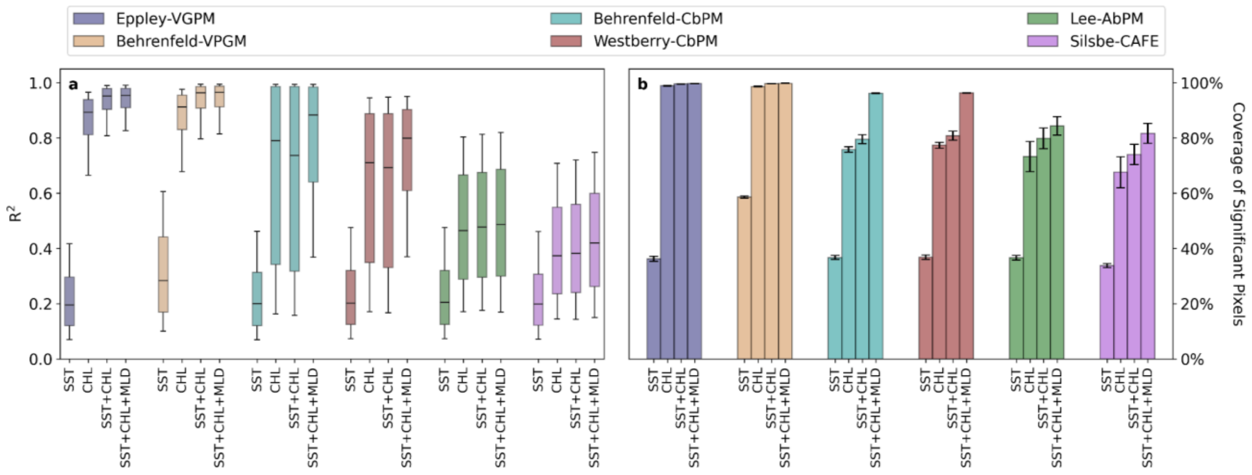

Figure S2: Sensitivity analysis of choice of metrics used in the multiple linear regression analyses. (a) Boxplots of R^2 values from multiple linear regression analyses using sea surface temperature (SST), chlorophyll-a concentrations (CHL), SST & CHL and mixed layer depth (MLD). (b) Bar chart of Jackknife mean±standard deviation % significant pixels ($p<0.05$) against choice of metrics. Multiple linear regression analyses performed using Jackknife resampling analysis on the Eppley-VGPM, Behrenfeld-VGPM, Behrenfeld-CbPM, Westberry-CbPM, Lee-AbPM and Silsbe-CAFE remote sensing NPP algorithms.

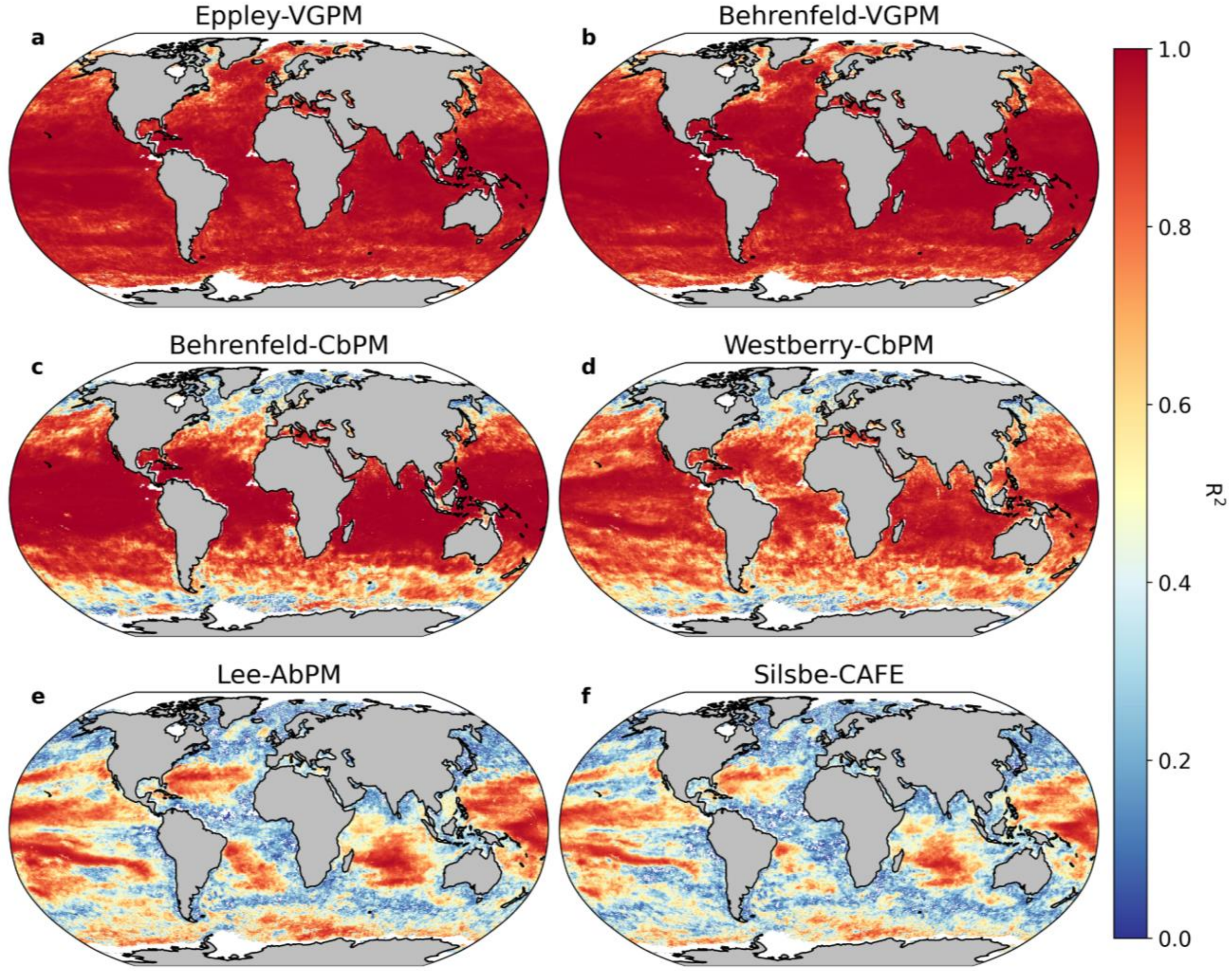

Figure S3: Spatial variability of multiple linear regression analyses performed on remote sensing models. Global maps of multiple linear regression adjusted R^2 values from the (a) Eppley-VGPM, (b) Behrenfeld-VGPM, (c) Behrenfeld-CbPM, (d) Westberry-CbPM, (e) Lee-AbPM and (f) Silsbe-CAFE NPP algorithms. Only pixels where the multiple linear regression model was significant ($p<0.05$) have been plotted in the maps, non-significant pixels ($p>0.05$) or pixels where a driver metric is missing from the time series are presented as white in the maps.

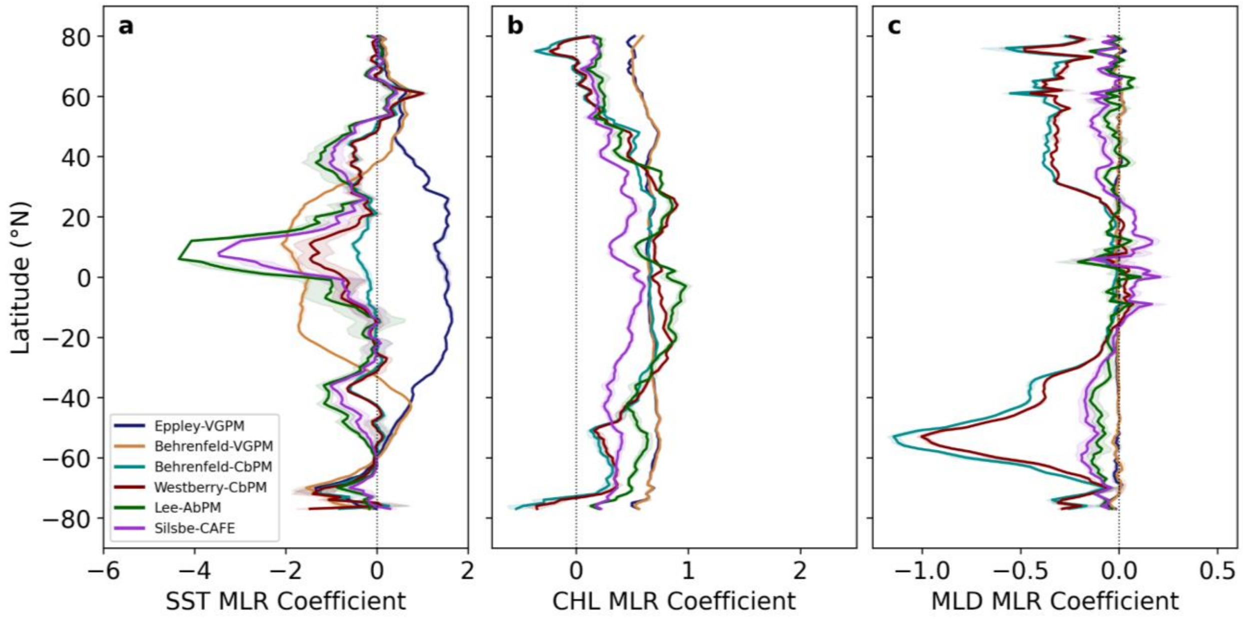

Figure S4: Comparing the Jackknife simulation variability of the multiple linear regression coefficients of the remote sensing algorithms. Jackknife simulation averages±standard deviations of the multiple linear regression coefficients for (a) sea surface temperature (SST), (b) chlorophyll-a concentrations (CHL), and (c) mixed layer depth (MLD) for the Eppley-VGPM, Behrenfeld-VGPM, Behrenfeld-CbPM, Westberry-CbPM, Lee-AbPM and Silsbe-CAFE NPP algorithms.

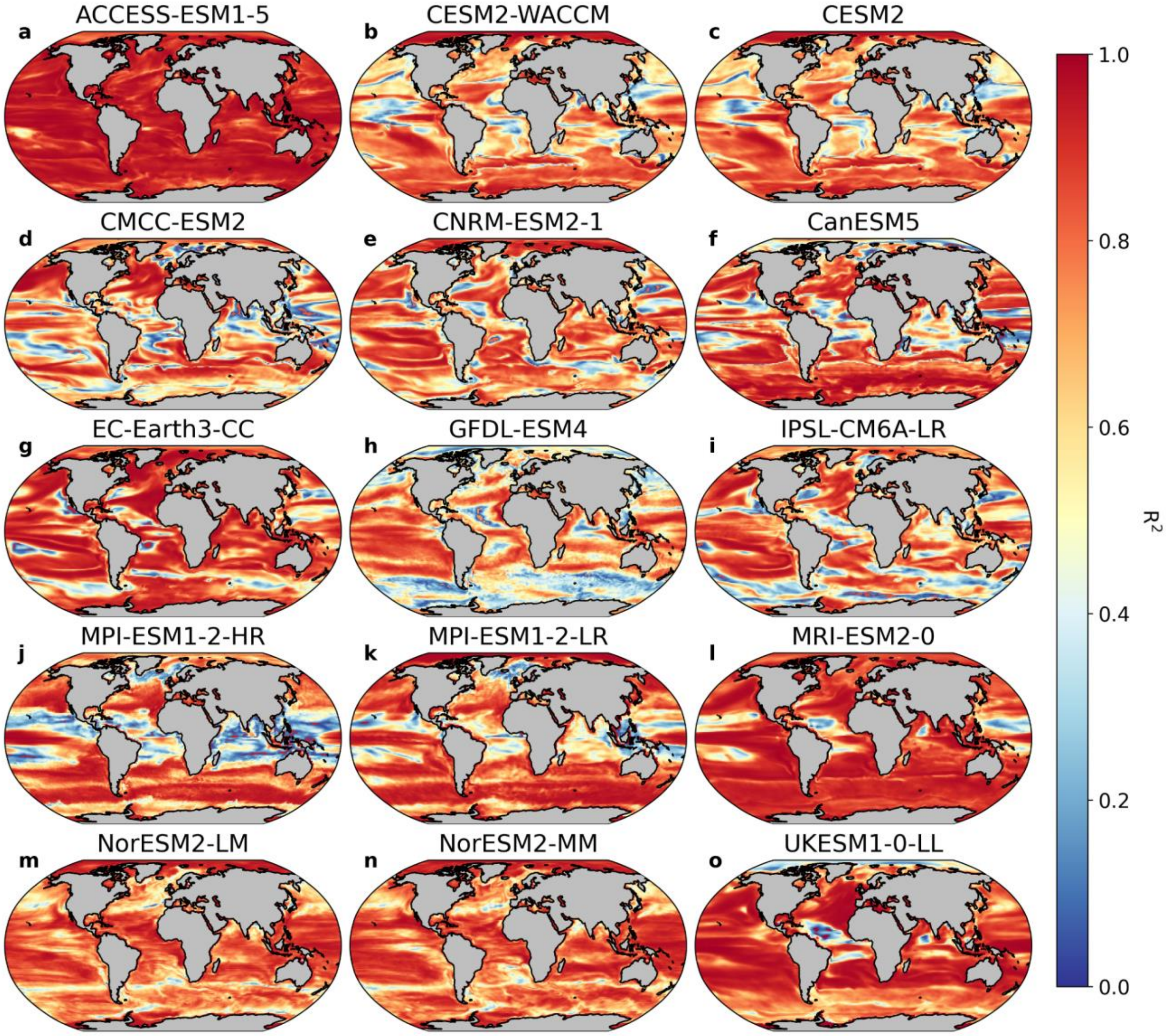

Figure S5: Spatial variability of multiple linear regression analyses performed on the CMIP6 Earth system models. Global maps of adjusted R^2 values from the (a) ACCESS-ECM1-5, (b) CESM2-WACCM, (c) CESM2, (d) CMMC-ESM2, (e) CNRM-ESM2-1, (f) CanESM5, (g) EC-Earth3-CC, (h) GFDL-ESM4, (i) IPSL-CM6A-LR, (j) MPI-ESM1-2-HR, (k) MPI-ESM1-2-LR, (l) MRI-ESM2-0, (m) NorESM2-LM, (n) NorESM2-MM and (o) UKESM1-0-LL. Only grid points where the multiple linear regression model was significant ($p<0.05$) have been plotted in the maps, non-significant grid points ($p>0.05$) or grid points where a driver metric is missing from the time series are presented as white in the maps.

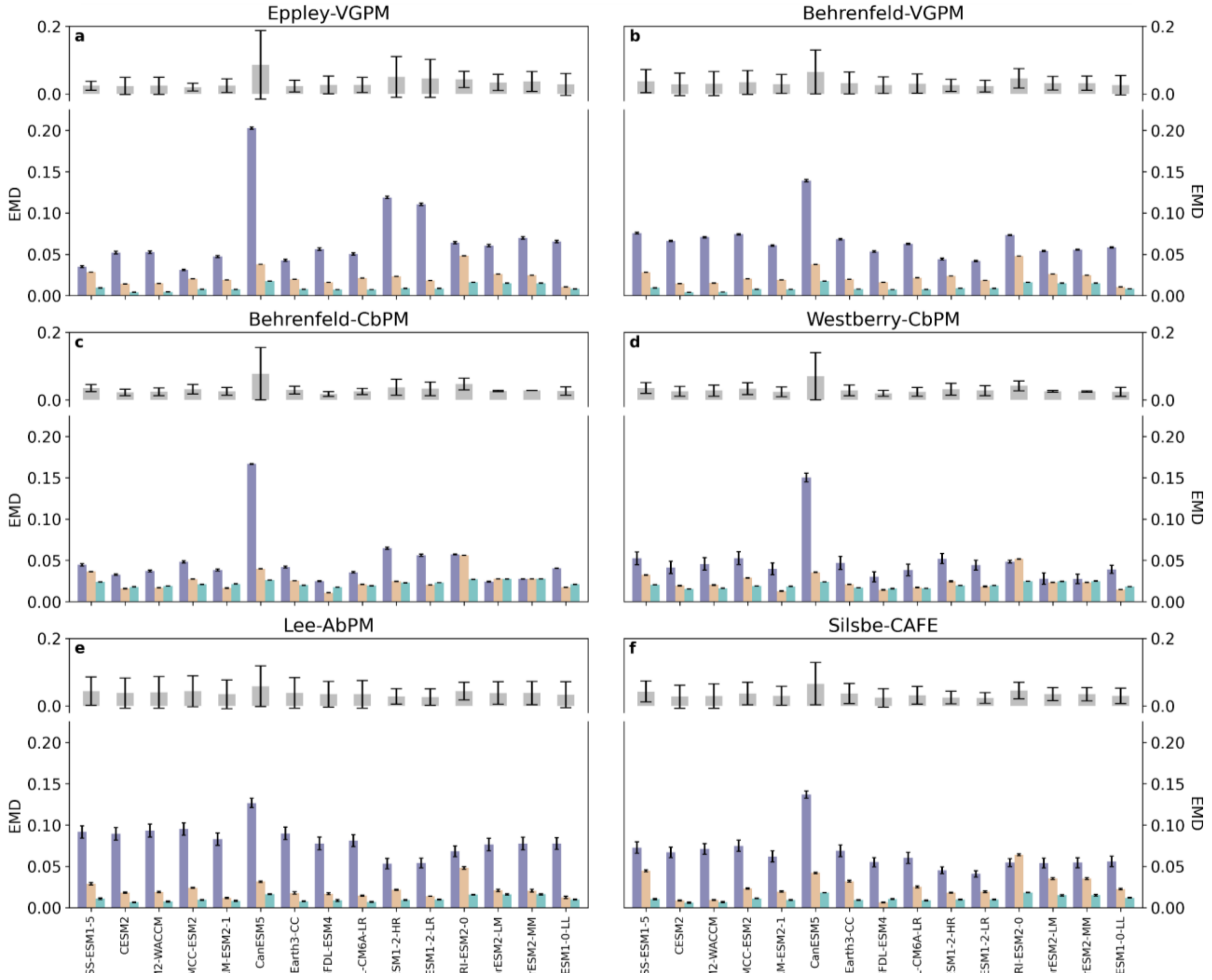

Figure S6: Comparing multiple linear regression coefficients from remote sensing algorithms to Earth system models. Bottom bar plots represent globally averaged biome-weighted Earth mover's distance (EMD) metric of multiple linear regression coefficients for sea surface temperature (SST), chlorophyll-a concentrations (CHL) and mixed layer depth (MLD) per Earth system model for the (a) Eppley-VGPM, (b) Behrenfeld-VGPM, (c) Behrenfeld-CbPM, (d) Westberry-CbPM, (e) Lee-AbPM and (f) Silsbe-CAFE NPP algorithms across all 7 Jackknife simulations. Top bar plots represent the mean±standard deviation across the metric drivers for each Earth system model per NPP algorithm. EMD metrics were calculated using multiple linear regression coefficients restricted using the IQR fence test (see methods) for both remote sensing and CMIP6 models.

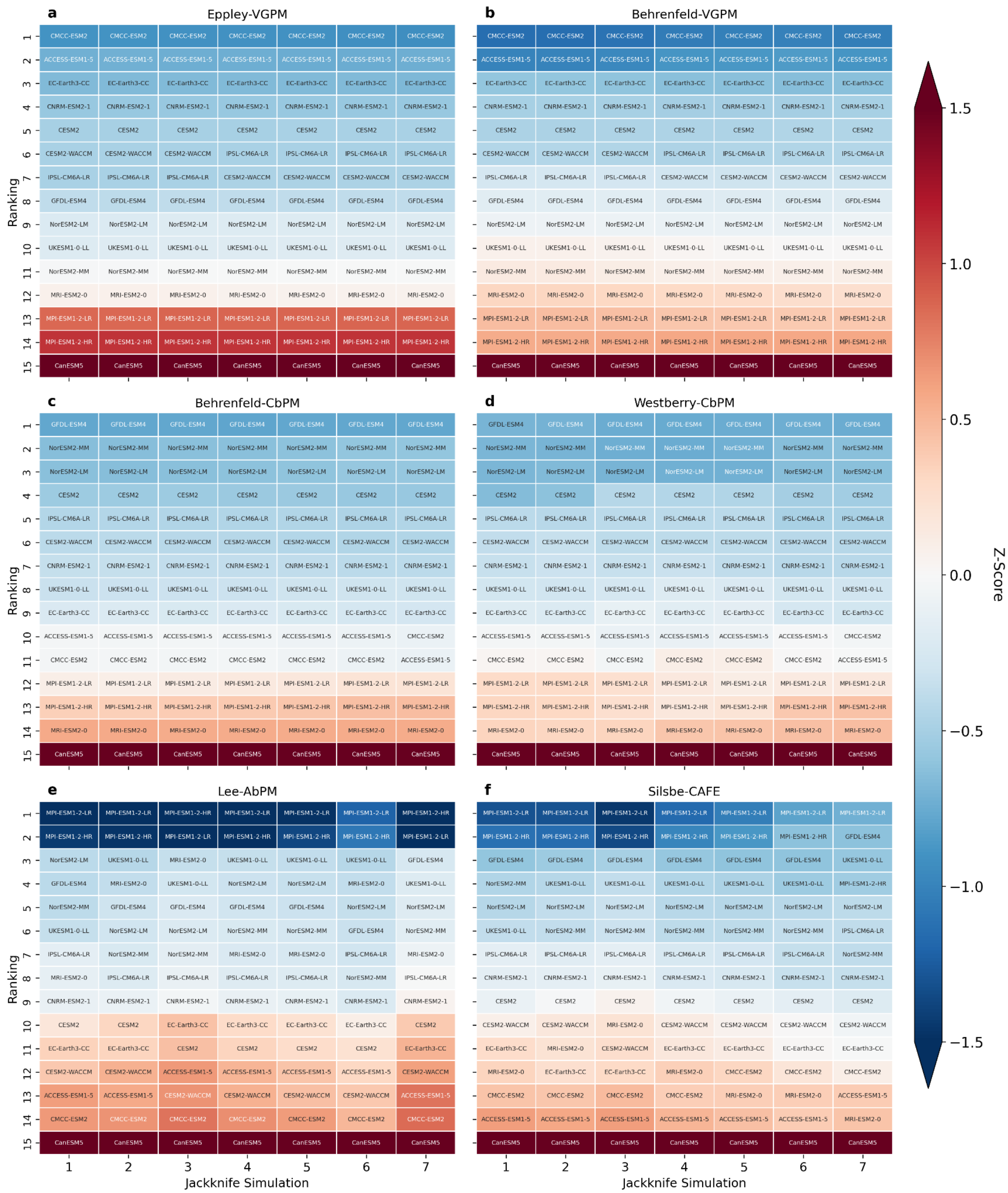

Figure S7: Ranking Earth system models using Z-score assessments of the Earth mover's distance metric for each Jackknife simulation. Heatmaps of Z-scores for ranked Earth system models per NPP remote sensing algorithm, including (a) Eppley-VGPM, (b) Behrenfeld-VGPM, (c) Behrenfeld-CbPM, (d) Westberry-CbPM, (e) Lee-AbPM and (f) Silsbe-CAFE.

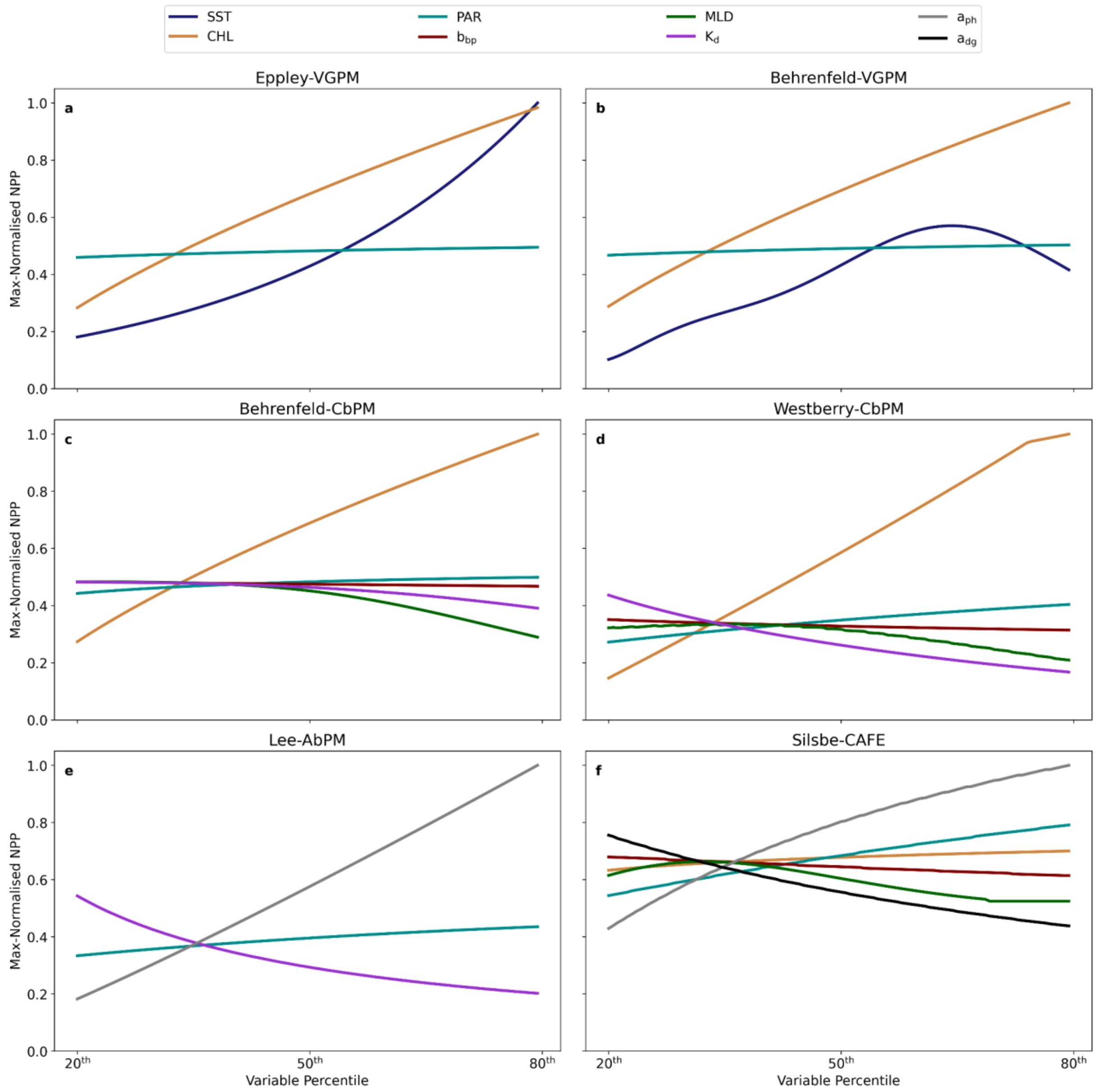

Figure S8: Exploring the input variable dependency in estimating net primary production. Line plots of max-normalised net primary production (NPP) calculated using the (a) Eppley-VGPM, (b) Behrenfeld-VGPM, (c) Behrenfeld-CbPM, (d) Westberry-CbPM, (e) Lee-AbPM and (f) Silsbe-CAFE NPP algorithms. Input variables include sea surface temperature (SST), chlorophyll-a (CHL), photosynthetically active radiation (PAR), particulate backscattering (b_{bp}), mixed layer depth (MLD), diffuse attenuation coefficient (K_d), phytoplankton absorption (a_{ph}) and detrital absorption (a_{dg}). The input variable being tested was allowed to range between the climatological (1998-2023) 20th and 80th percentile, whilst the other input variables were held constant at the climatological median value.

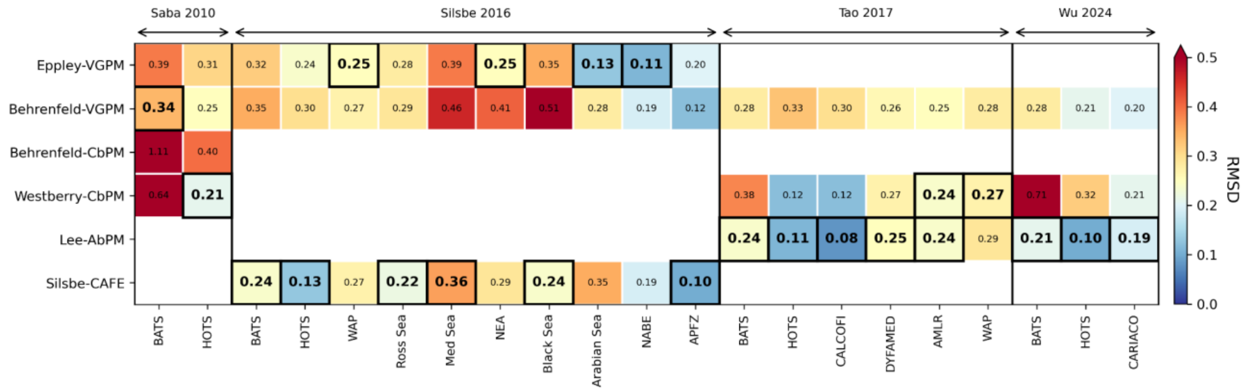

Figure S9: Comparing differences between remote sensing NPP algorithms and direct field measurements. Root mean square differences (RMSD) between NPP estimated from Eppley-VGPM, Behrenfeld-VGPM, Behrenfeld-CbPM, Westberry-CbPM, Lee-AbPM and Silsbe-CAFE NPP algorithms and direct field measurements from the Bermuda Atlantic Time Series (BATS), Hawaii Oceanic Time Series (HOTS), Western Antarctic Peninsula (WAP), coastal north east Atlantic (NEA), Black Sea, Arabian Sea, pelagic North Atlantic (NABE), Antarctic Polar Frontal Zone (APFZ), California coast (CALCOFI), Mediterranean Sea (DYFAMED), Scotia Sea (AMLR), Cariaco basin (CARIACO)[21,32–34]. Cells with bold text and a black border represent the algorithm which had the lowest RMSD for the specific study. Please note that empty cells means that the algorithm was not implemented during the study.

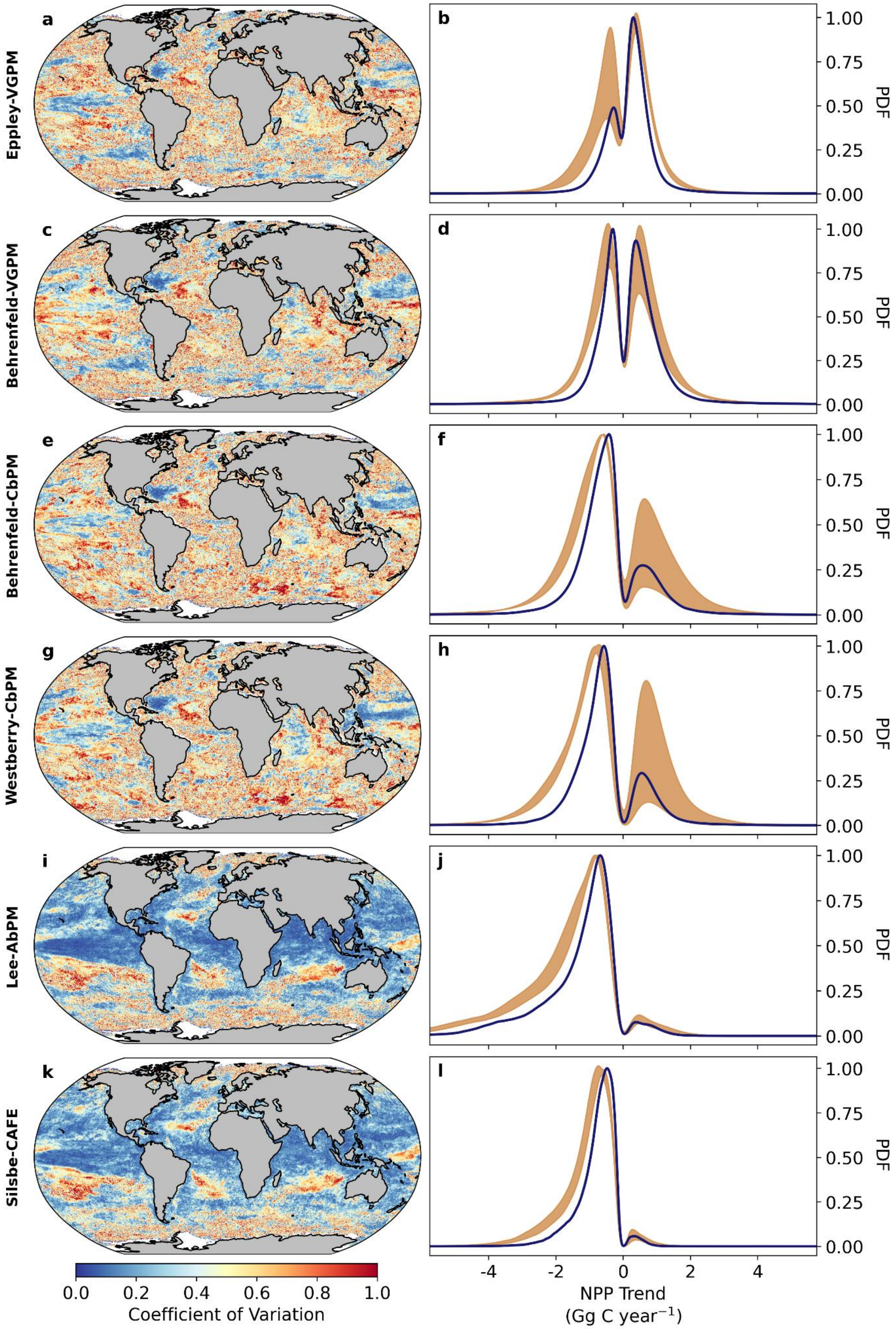
a
b
c
d
e
f
g
h
i
j
k
l
Eppley-VGPM
Behrenfeld-VGPM
Behrenfeld-CbPM
Westberry-CbPM
Lee-AbPM
Silsbe-CAFE
PDF
0.00
0.25
0.50
0.75
1.00
-4
-2
0
2
4
NPP Trend
(Gg C year^{-1})
0.0
0.2
0.4
0.6
0.8
1.0
Coefficient of Variation

90

Figure S10: Exploring the sensitivity of trends in annual mean net primary production. Maps of coefficient of variation (a,c,e,f,g,l,k) and normalised probability density function (PDF) plots (b,d,f,h,j,l) of trends in annual mean net primary production (NPP; Gg year^{-1}) from 1998-2023 (Original) and the results from a Monte Carlo Jackknife experiment in which 20 years of the 1998-2023 period are sub-sampled for trend calculations (Jackknife Simulations) for the (a,b) Eppley-VGPM, (c,d) Behrenfeld-VGPM, (e,f) Behrenfeld-CbPM, (g,h) Westberry-CbPM, (i,j) Lee-AbPM and (k,l) Silsbe-CAFE NPP algorithms. Coefficient of variation calculated as the Jackknife trend 1σ over the absolute mean Jackknife trends. Shaded regions in the PDF plots represent the Jackknife Simulation mean±standard deviation. Only pixels where the trend is significant ($p<0.05$) are included in the PDF distributions.

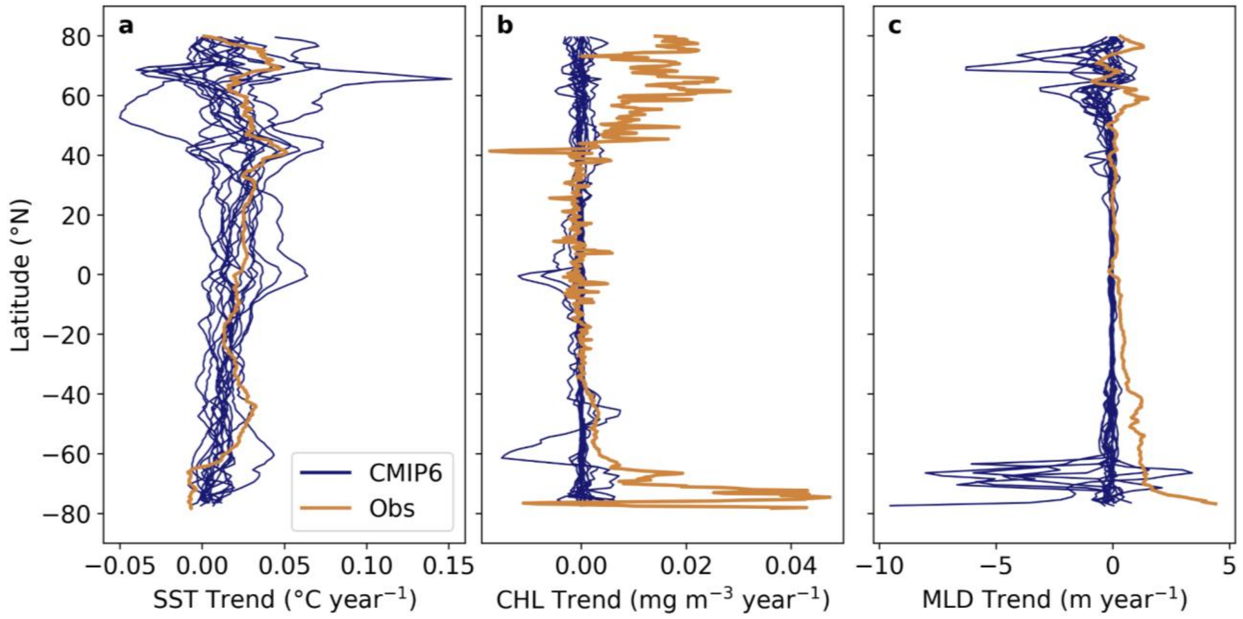

Figure S11: Spatial variability of the driver annual trends. Zonal averages±standard deviations of the annual trends for (a) sea surface temperature (SST), (b) chlorophyll-a concentrations (CHL) and (c) mixed layer depth (MLD) for the ensemble of CMIP6 Earth system models and observations.
